# Association between proteinuria trajectories and outcomes in critically ill patients with sepsis or shock

**DOI:** 10.1371/journal.pone.0272835

**Published:** 2022-08-24

**Authors:** Raphael Monge, Charlotte Oris, Matthieu Jabaudon, Marina Braïlova, Emmanuel Futier, Vincent Sapin, Bruno Pereira, Alexandre Lautrette

**Affiliations:** 1 Department of Perioperative Medicine, CHU Clermont-Ferrand, Clermont-Ferrand, France; 2 Department of Medical Biochemistry and Molecular Genetics, CHU Clermont-Ferrand, Clermont-Ferrand, France; 3 GReD, Université Clermont Auvergne, CNRS, INSERM, Clermont-Ferrand, France; 4 Biostatistics Unit, Department of Clinical Research and Innovation (DRCI), CHU Clermont-Ferrand, Clermont-Ferrand, France; 5 Department of Intensive Care Medicine, CHU Clermont-Ferrand, Clermont-Ferrand, France; 6 LMGE (Laboratoire Micro-organismes: Génome et Environnement), UMR CNRS 6023, Université Clermont Auvergne, Clermont-Ferrand, France; PLOS (Public Library of Science), UNITED KINGDOM

## Abstract

**Background:**

Proteinuria results from kidney damage and can be a predictor of illness severity and mortality in the intensive care unit (ICU). However, the optimal timing of proteinuria measurements and the reference values remain undetermined. Our objective was to identify the patterns of proteinuria change associated with mortality in ICU patients with sepsis or shock.

**Methods:**

This monocentric retrospective cohort study performed from April 2010 to April 2018 involved all ICU patients with sepsis or shock and at least two measurements of proteinuria from a 24h-urine collection during the first 10 days of ICU stay, the first of which was made within 48h after ICU admission. We identified proteinuria trajectories by a semi-parametric mixture model and analysed the association between the trajectories and the mortality at day 28 by Cox proportional-hazards model.

**Results:**

A total of 3,344 measurements of proteinuria from 659 patients were analysed. Four proteinuria trajectories were identified. Trajectories 1, 2, 3 and 4 comprised 127, 421, 60 and 51 patients, and were characterized by a first proteinuria of 1.14 [0.66–1.55], 0.52 [0.26–0.91], 2.92 [2.38–3.84] and 2.58 [1.75–3.32] g/24h (p<0.001) and a mortality of 24.4%, 38%, 20% and 43% (p = 0.002), respectively. Trajectories 3 and 4 had a high first proteinuria (>2g/24h). Only, the proteinuria of trajectory 4 increased within 3 days following the first measurement and was associated with increased mortality at day 28 (hazard ratio: 2.36 95%CI [1.07–5.19], p = 0.03), regardless of acute renal failure. The factors associated with trajectory 4 were cancer (relative risk: 8.91 95%CI [2.09–38.02], p = 0.003) and use of inotropic drugs (relative risk: 0.17 95%CI [0.04–0.69], p = 0.01).

**Conclusion:**

This exploratory study of ICU patients with sepsis or shock identified four proteinuria trajectories with distinct patterns of proteinuria change over time and mortality rates. These results provide novel insights into renal pathophysiology and may be helpful to investigate subphenotypes of kidney injury among ICU patients in future studies.

## 1. Introduction

Proteinuria is the consequence of glomerular and/or tubular damage. The host responses that characterize the critical illness are associated with changes in glomerular hemodynamics and pore size that result in transient proteinuria [1]. A study on a series of post-mortem human kidney biopsies showed that renal histopathology during a septic shock involved acute tubular injury accompanied by glomerular lesions including capillary leukocytic infiltration, apoptosis and thrombi [2]. Transient, sometimes intense, proteinuria has been reported in certain stress states such as fever, exercise [3], severe burns, sepsis, shock [4–6] and more recently during COVID-19 [7, 8]. High proteinuria (>2g/24h) is typically caused by glomerular lesions [9]. Glomerular proteinuria results from endothelial and/or podocyte dysfunction that leads to increased vascular permeability of the glomerular filtration barrier [10]. Some experimental studies have suggested systemic pro-inflammatory cytokines are involved in the process of glomerular lesions during sepsis or shock [6, 11]. Proteinuria can be independent of acute kidney injury (AKI) and be detected before a potential decrease in the glomerular filtration rate [12, 13]. Some studies have reported that glomerular proteinuria, like albuminuria, is a predictor of illness severity and mortality in critically ill patients [14], independently of AKI. However, the optimal timing of the measurements of proteinuria and the reference values are still undetermined.

This exploratory study aimed to identify the proteinuria trajectories associated with mortality in ICU patients with major inflammatory states including sepsis and shock. The secondary objective was to describe the patient characteristics associated with these trajectories.

## 2. Methods

### 2.1 Study design

We conducted a retrospective cohort study of adult patients (≥ 18 years) admitted to an ICU of the Clermont-Ferrand University Hospital in France between 1 April 2010 and 1 April 2018. Inclusion criteria were sepsis or shock and at least two measurements of proteinuria from a 24h-urine collection during the first 10 days of ICU stay, the first of which was performed within 48h after ICU admission. Patients were identified from the French national hospital database (Program for Medicalization of Information Systems) with the International Classification of Diseases diagnostic codes for “sepsis” and “shock” and the hospital database MIPS/Glims^®^ for the values of proteinuria. The diagnoses of sepsis and septic shock were checked to meet current definitions [15]. The diagnosis of cardiogenic and hypovolemic/haemorrhagic shock was checked to meet the use of inotropic and vasopressor drug, respectively. Exclusion criteria were the occurrence of end-stage renal failure requiring renal replacement therapy and the occurrence of urinary tract infection or macroscopic haematuria during the first 10 days of ICU stay. The values of proteinuria associated with an oligo-anuria defined as urine output less than 100ml/24h, were excluded from the statistical analyses. Patients or relatives were notified during the hospital stay that data would be extracted from medical records for research purposes. They had the opportunity of participation refusal. All data were fully anonymized before we accessed them. The study was approved by the Southeast People’s Protection Committee (Comité de Protection des Personnes Sud-Est, reference # 2018/CE30 –N°IRB00008526) and was conducted according to the STROBE guidelines regarding observational cohort studies (S1 Checklist).

### 2.2 Data collection

Patient characteristics, biological data and the outcome at day 28 from ICU admission were extracted from the patient’s electronic medical record. Patient characteristics included age, sex, weight and body mass index (BMI) at ICU admission, simplified acute physiology score (SAPS) II at ICU admission, the main major inflammatory state including shock (septic or cardiogenic or hypovolemic/haemorrhagic) or sepsis, chronic kidney disease [16], chronic heart failure [17], hypertension requiring treatment, diabetes requiring treatment, uncured cancer, cirrhosis [18], chronic treatment with renin-angiotensin-aldosterone system blockers, invasive and non-invasive mechanical ventilation, AKI based on the creatinine criteria [19], AKI requiring renal replacement therapy, diuretics in ICU, vasopressor, and inotropic drugs. The proteinuria from a 24h-urine collection was measured at 08:00 AM by the pyrogallol red-molybdate method on the Dimension Vista^®^ 1500 system (Siemens Healthcare Diagnostics Inc). This method measures total urinary protein with a minimum threshold of 50mg/L but the nature of protein is not identified. The days of measurement were not the same between the patients.

### 2.3 Statistical analysis

Statistical analyses were performed using Stata software, Version 15 (StataCorp, College Station, US). All tests were two-sided, with a Type I error set at 5%. Continuous data were expressed as mean ± standard-deviation or median [interquartile range] according to statistical distribution. The assumption of normality was assessed by the Shapiro-Wilk test. To analyse longitudinal data (i.e. proteinuria change), random-effects models for repeated data were performed, with time as fixed effect and patient as random-effect to take into account between and within patient variability. To compare changes between groups, group x time-point evaluation interaction was studied. A Sidak’s type I error correction was applied to perform multiple comparisons. The normality of residuals was studied with the Shapiro-Wilk test.

To identify distinctive trajectories of proteinuria, semi-parametric mixture models (group-based trajectory model) were carried out to model the relationship between proteinuria and time for each trajectory, the shape of the trajectory, and the estimated proportion of the population belonging to each trajectory. These probabilities are called the posterior probability of group membership. To create the profiles, individuals were assigned to the trajectory group to which they most likely belonged based on their measured history of proteinuria. Groupings can identify distinct subpopulations. The analysis provides a formal way to determine the best-fit number of trajectories and a precise estimate of group membership allocation, which can be expressed using observed probabilities and posteriori probabilities. Nagin lays out several statistically oriented criteria for assessing model adequacy [20]. These include: (a) obtaining for each trajectory group a close correspondence between the estimated probability of group membership and the proportion assigned to that group based on the posterior probability of group membership, (b) ensuring that the average of the posterior probabilities of group membership for individuals assigned to each group exceeds a minimum threshold of 0.7, (c) establishing that the odds of correct classification based on the posterior probabilities of group membership exceed a minimum threshold of 5, and (d) observing reasonably tight confidence intervals around estimated group membership probabilities. The best-fitting model is selected according to the Bayesian Information Criterion.

The continuous variables were then compared between independent trajectory groups by ANOVA, or Kruskal–Wallis test if the assumptions of ANOVA were not met. Homoscedasticity was analysed with the Bartlett test. Comparisons between independent trajectories were carried out with Chi-squared or Fisher’s exact tests for categorical variables. To determine the parameters associated with proteinuria trajectories, multivariable analyses (i.e. multinomial polytomous logistic regression) were carried out using the stepwise approach (backward and forward) on covariates fixed according to univariable results and to clinical relevance: gender, age, cancer, inotropic drugs, AKI stages, SAPSII, major inflammatory state, renal replacement therapy, mechanical ventilation, vasopressor drugs. Particular attention was paid to the study of multicollinearity and interactions between covariates by studying the relationships between the covariates, and evaluating the impact of adding or deleting variables on the multivariable model. Results were expressed as relative risks (RR) and 95% confidence intervals (CI).

Estimates of overall survival at 28 days were constructed with the Kaplan-Meier method. Cox proportional hazards regression model was used to investigate associated prognostic factors in univariable and multivariable analysis. For multivariable analysis, the covariates were determined according to univariable results and to the clinical relevance with a particular attention paid to multicollinearity. The proportional-hazard hypothesis was verified using Schoenfeld’s test and plotting residuals. Results were expressed as hazard-ratio (HR) and 95% confidence interval.

Sensitivity analyses were carried out to evaluate the impact of missing data on results. The above analyses were conducted for patients with at least three values of proteinuria during the first 10 days of ICU stay, including the first measurement. Furthermore, an imputation data approach was also applied on proteinuria values taking into account their not missing not at random statistical nature, principally due to values censored by death. Multiple imputation by the two-fold fully conditional specification algorithm was performed imputing missing values at each time point conditional on observed measurements within a small time window using chained equations.

## 3. Results

Over the study period, we identified 1217 patients admitted to the ICU with sepsis or shock. A total of 659 patients met the inclusion criteria of proteinuria measurements and formed the study population (S1 Fig). Their characteristics are shown in Table 1.

**Table 1:** Characteristics of the study population.

Variables	Study Population
Patients, n	659
Male gender, n (%)	442 (67)
Age, years	65 [56–75]
Chronic kidney disease, n (%)	31 (4.7)
Hypertension, n (%)	112 (17.0)
Chronic heart failure, n (%)	81 (12.3)
Cancer, n (%)	104 (15.8)
Cirrhosis, n (%)	50 (7.6)
Diabetes, n (%)	55 (8.4)
Renin-angiotensin-aldosterone system blockers, n (%)	38 (5.8)
Weight at ICU admission, kg	75 [65–88]
BMI at ICU admission, kg/m^2^	26 [23–30]
SAPS II at ICU admission, points	55 [44–66]
Major inflammatory state, n (%)	
Septic shock	369 (56.0)
Cardiogenic shock	60 (9.1)
Hypovolemic/Haemorrhagic shock	146 (22.2)
Sepsis	84 (12.7)
Invasive mechanical ventilation, n (%)	533 (80.9)
Non-invasive mechanical ventilation, n (%)	286 (43.4)
Vasopressor drugs, n (%)	548 (83.2)
Inotropic drugs, n (%)	119 (18.1)
Diuretics in ICU, n (%)	430 (65.3)
Serum creatinine at day 1, μmol/L	115 [74–193]
Serum creatinine at day 10, μmol/L	81 [60–118]
Acute kidney injury, n (%)	
Stage 1	184 (27.9)
Stage 2	65 (9.9)
Stage 3	214 (32.5)
Acute kidney injury requiring renal replacement therapy, n (%)	179 (27.2)
Minimum level of PO_2_/FiO_2_ ratio during the first 10 days	163 [104–248]
Maximum level of total bilirubin during the first 10 days, μmol/L	17 [10–39]
First proteinuria, g/24h	0.78 [0.37–1.47]
Length of ICU stay, days	10 [6–20]
ICU mortality, n (%)	213 (32.3)
Mortality at day 28, n (%)	225 (34.2)

BMI: body mass index; SAPS II: simplified acute physiology score 2

### 3.1 Identification of proteinuria trajectories

**Fig 1:**
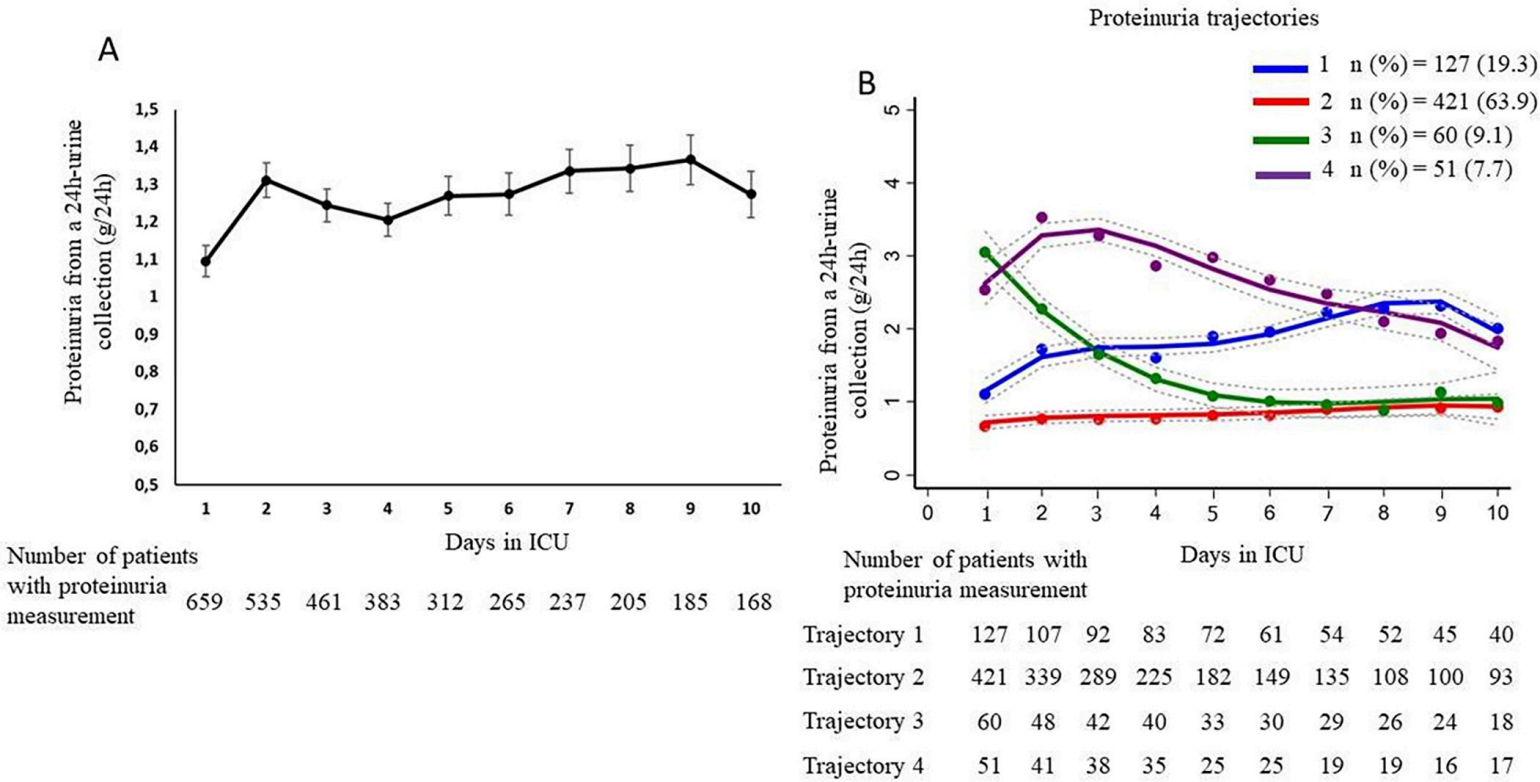
Change of proteinuria within the first 10 days in ICU. A. Mean proteinuria of the study population within the first 10 days in ICU. B. Proteinuria trajectories during the first 10 days in ICU. blue curve: trajectory 1; red curve: trajectory 2; green curve: trajectory 3; purple curve: trajectory 4. The dotted lines correspond to the standard deviations.

A total of 3,344 proteinuria measurements collected over the first 10 days of ICU stay were analysed. During this period, the means of proteinuria measurements per patient for trajectories 1, 2, 3 and 4 were 5.7 ± 3.0, 4.7 ± 2.7, 5.7 ± 3.0 and 5.4 ± 2.8, respectively. The change in the mean proteinuria level of the study population during these 10 ICU days is shown in Fig 1A.

**Fig 2:**
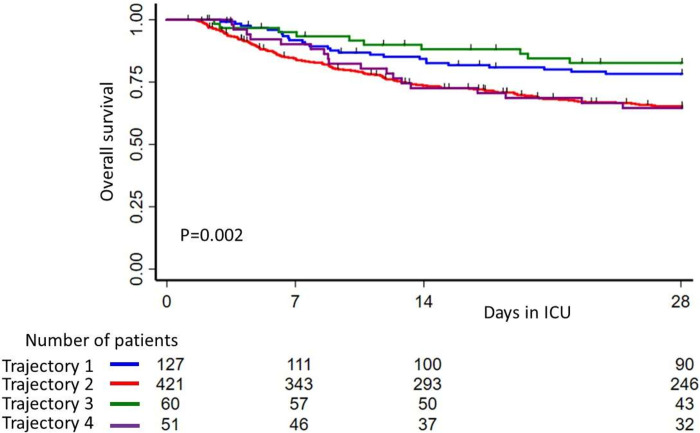
Survival probability within 28 days after ICU admission. blue curve: trajectory 1; red curve: trajectory 2; green curve: trajectory 3; purple curve: trajectory 4.

Using the group-based trajectory model approach, we identified four proteinuria trajectories (Fig 1B). Trajectories 1 and 2 comprised 127 (19.3%) and 421 (63.9%) patients respectively, who had a low first proteinuria level of 1.14 [0.66–1.55] g/24h and 0.52 [0.26–0.91] g/24h. Trajectory 1 had a proteinuria level that increased over the 10 days up to 2.05 [1.35–2.45] g/24h while those of trajectory 2 remained low at 0.88 [0.49–1.18] g/24h (Fig 2B). Trajectories 3 and 4 comprised 60 (9.1%) and 51 (7.7%) patients respectively, who had a high first proteinuria level of 2.92 [2.38–3.84] g/24h and 2.58 [1.75–3.32] g/24h (p = 0.12). Trajectory 3 had proteinuria levels that decreased continuously on day 3 (1.56 [1.11–2.25] g/24h, p<0.001) and over the 10 days to 0.83 [0.55–1.31] g/24h (first vs day 10 proteinuria, p = 0.001). Proteinuria levels of trajectory 4 increased over the first three days to 3.13 [2.65–3.98] g/24h (p = 0.03) and then decreased to 1.57 [1.28–1.2.12] g/24h on day 10 (first vs day 10 proteinuria, p = 0.09) (Fig 2B). Proteinuria levels between trajectories 3 and 4 were significantly different on day 3 (p<0.001) and day 10 (p<0.001). The criteria assessing model adequacy were i) posterior probabilities for the trajectories 1, 2, 3, and 4 equal to 20.4%, 62.3%, 9.6% and 7.7%, respectively, ii) average posterior probabilities of 85.5%, 93.6%, 87.9% and 93.2%, respectively (expected >70%), and iii) odds of correct classification based on the posterior probabilities of group membership: 24.8, 8.2, 72.6 and 163.3, respectively (expected >5).

A sensitivity analysis that included only the patients with at least three proteinuria measurements during the first 10 days of ICU stay (n = 434 patients) and another sensitivity analysis performed with the study population and data imputed by multiple imputation by the two-fold fully conditional specification algorithm were conducted to verify the robustness of the model in identifying trajectories and determined four very similar trajectories (S2 and S3 Figs).

### 3.2 Association between mortality and proteinuria trajectories

Comparison of the patient characteristics associated with the trajectories showed differences in the age and proportion of men, cancer, cirrhosis and patients receiving inotropic drugs (Table 2). There was a significant difference overall in the first proteinuria levels between the four trajectories, but which was not found between trajectories 3 and 4 (p = 0.12). There was also a significant difference between mortalities at day 28 in the four trajectories. A sensitivity analysis that excluded the patients with only sepsis, found similar results (data not shown).

**Table 2:** Comparison of patient characteristics associated with the four trajectories.

Variables	Population of trajectory 1	Population of trajectory 2	Population of trajectory 3	Population of trajectory 4	Omnibus p-value
Patients, n (%)	127 (19.3)	421 (63.9)	60 (9.1)	51 (7.7)	
Proteinuria measurements, n (%)	724 (21.6)	2003 (59.9)	340 (10.2)	277 (8.3)	
Male gender, n (%)	75 (59.1)	280 (66.5)	46 (76.7)	41 (80.4)	0.02
Age, years	65 [55–76]	67 [57–76]	64 [50–73]	62 [52–67]	0.02
Chronic kidney disease, n (%)	5 (3.9)	20 (4.8)	3 (5.0)	3 (5.9)	0.91
Hypertension, n (%)	21 (16.5)	70 (16.3)	12 (20.0)	9 (17.7)	0.93
Chronic heart failure, n (%)	11 (8.7)	56 (13.3)	6 (10.0)	8 (15.7)	0.43
Cancer, n (%)	19 (15.0)	64 (15.2)	5 (8.3)	16 (31.4)	0.01
Cirrhosis, n (%)	2 (1.6)	42 (10.0)	5 (8.3)	1 (2.0)	0.01
Diabetes, n (%)	8 (6.3)	38 (9.0)	5 (8.3)	4 (7.8)	0.84
Renin-angiotensin-aldosterone system blockers, n (%)	11 (8.7)	21 (5.0)	3 (5.0)	3 (5.9)	0.45
Weight at ICU admission, kg	75 [67–87]	75 [65–85]	80 [72–96]	75 [68–90]	0.09
BMI at ICU admission, kg/m^2^	26 [23–30]	26 [23–31]	27 [24–32]	25 [22–28]	0.51
SAPS II at ICU admission, points	55 [42–65]	55 [44–67]	57 [44–66]	55 [44–65]	0.54
Major inflammatory state, n (%)					0.17
Septic shock	77 (60.6)	225 (53.4)	35 (58.3)	32 (62.7)	
Cardiogenic shock	10 (7.9)	43 (10.2)	6 (10.0)	1 (2.0)	
Hypovolemic/Haemorrhagic shock	24 (18.9)	104 (24.7)	7 (11.7)	11 (21.6)	
Sepsis	16 (12.6)	49 (11.7)	12 (20.0)	7 (13.7)	
Invasive mechanical ventilation, n (%)	100 (78.7)	347 (82.4)	48 (80.0)	38 (74.5)	0.50
Non-invasive mechanical ventilation, n (%)	64 (50.4)	172 (40.9)	29 (48.3)	21 (41.2)	0.23
Vasopressor drugs, n (%)	103 (81.1)	350 (83.1)	53 (88.3)	42 (82.4)	0.67
Inotropic drugs, n (%)	13 (10.2)	86 (20.4)	14 (23.3)	6 (11.8)	0.03
Diuretics in ICU, n (%)	88 (69.3)	269 (63.9)	41 (68.3)	32 (62.8)	0.65
Serum creatinine at day 1, μmol/L	109 [76–192]	113 [72–188]	133 [78–209]	153 [70–213]	0.62
Serum creatinine at day 10, μmol/L	82 [66–102]	82 [60–120]	72 [51–113]	93 [53–143]	0.93
Acute kidney injury, n (%)					0.40
Stage 1	42 (33.1)	111 (26.4)	17 (28.3)	14 (27.4)	
Stage 2	8 (6.3)	41 (9.7)	11 (18.3)	5 (9.8)	
Stage 3	37 (29.1)	142 (33.7)	17 (28.3)	18 (35.3)	
Acute kidney injury requiring renal replacement therapy, n (%)	28 (22.1)	124 (29.4)	15 (25.0)	12 (23.5)	0.35
Minimum level of PO_2_/FiO_2_ ratio during the first 10 days	163 [111–243]	164 [103–250]	140 [89–213]	176 [125–245]	0.34
Maximum level of total bilirubin during the first 10 days, μmol/L	15 [9–29]	20 [10–47]	14 [10–25]	19 [11–44]	0.05
First proteinuria, g/24h	1.14 [0.66–1.55]	0.52 [0.26–0.91]	2.92 [2.38–3.84]	2.58 [1.75–3.32]	<0.001
Length of ICU stay, days	10 [6–29]	9 [5–19]	11 [8–22]	10 [6–19]	0.06
ICU mortality, n (%)	30 (23.6)	150 (35.6)	11 (18.3)	22 (43.1)	0.002
Mortality at day 28, n (%)	31 (24.4)	160 (38.0)	12 (20.0)	22 (43.1)	0.002

BMI: body mass index; SAPS II: simplified acute physiology score 2

**Fig 3:**
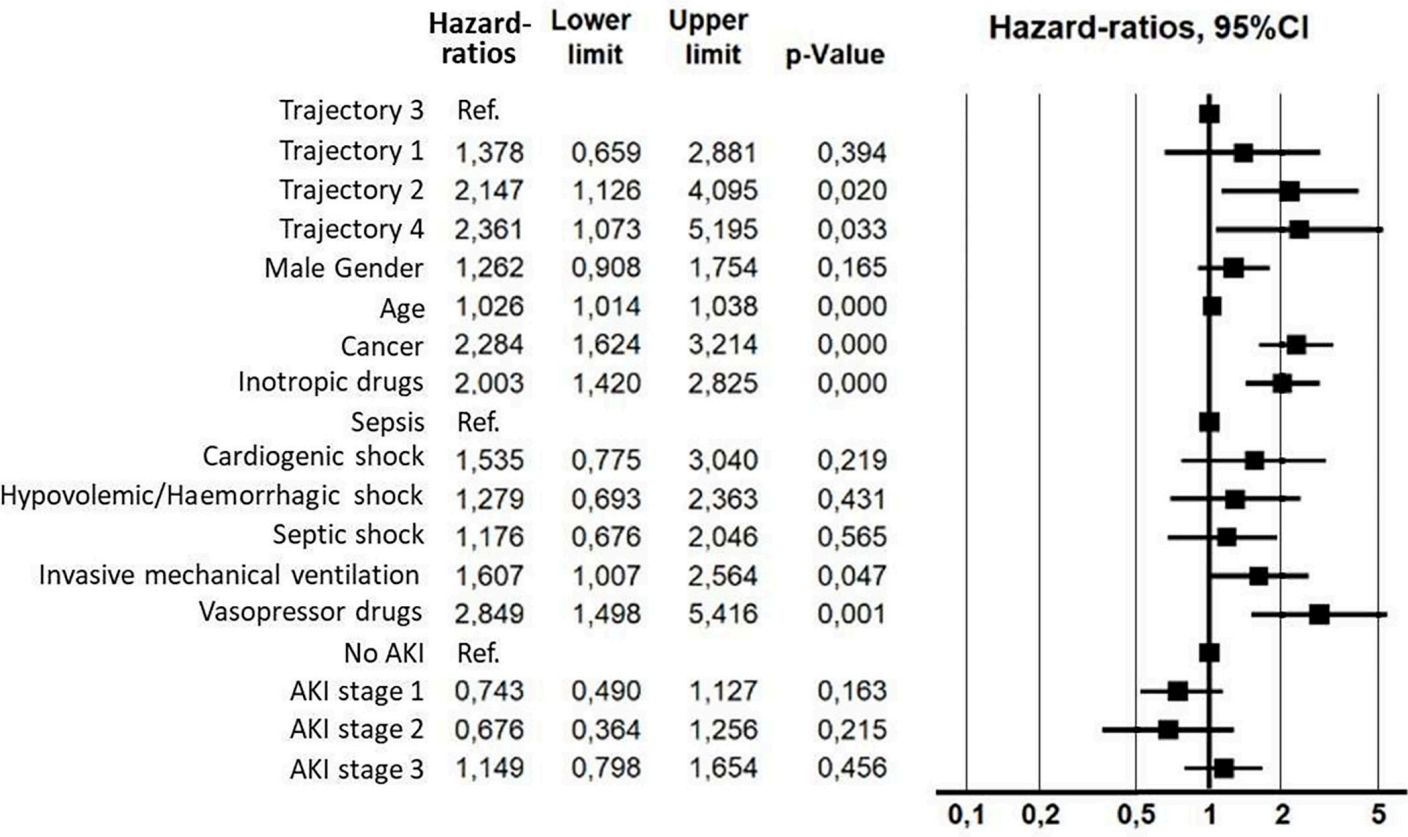
Factors associated with mortality (multivariable analysis). AKI: Acute Kidney Injury.

The survival curve at day 28 of trajectory 4 was the lowest and statistically different from that of trajectory 3 (p = 0.038) but not from the other two trajectories (trajectory 1, p = 0.068 and trajectory 2, p = 0.93) (Fig 2). Factors associated with mortality in the univariable analysis are given in S1 Table. In multivariable analysis (Fig 3), mortality was associated with trajectory 2 (hazard ratio (HR): 2.15 95%CI [1.13–4.09], p = 0.02), trajectory 4 (HR: 2.36 95%CI [1.07–5.19], p = 0.03), age (HR: 1.03 95%CI [1.01–1.04], p = 0.001), cancer ((HR: 2.28 95%CI [1.62–3.21], p = 0.001), inotropic drugs (HR: 2.00 95%CI [1.42–2.82], p = 0.001), invasive mechanical ventilation (HR: 1.61 95%CI [1.01–2.56], p = 0.047) and vasopressors (HR: 2.85 95%CI [1.50–5.42], p = 0.001).

### 3.3 Identification of the patient characteristics associated with the trajectories

Multivariable analysis was carried out using multinomial regression to identify the patient characteristics associated with the trajectories. We focused on the comparison between trajectories 3 and 4, because although they both had high first proteinuria levels, the outcomes (mortality rate) were different. The factors associated with the trajectory 4 were cancer (relative risk (RR): 8.91 95%CI [2.09–38.02], p = 0.003) and use of inotropic drugs (RR: 0.17 95%CI [0.04–0.69], p = 0.01) compared to trajectory 3 (S2 Table).

## 4. Discussion

This exploratory observational study found that a high first proteinuria level, close to 2 g/24 h, in ICU patients with shock or sepsis, is associated with an increased risk of death on day 28 if the level had increased in the first 3 days of the ICU stay, regardless of acute kidney injury. An association between a proteinuria trajectory and ICU mortality has not previously been reported.

There are few reports in the literature dealing with proteinuria in critically ill patients. Experimental studies reported that cytokines transiently alter the capillary glomerular barrier, thus allowing the transfer of blood proteins into the urine. The concept of organ damage caused by cytokines from other organs during the aggression phase is a widely documented occurrence, particularly interactions between the kidney and the lung [21–23]. Several studies have shown that hypoxemic respiratory failure, especially during ARDS, stimulates inflammatory pathways in the kidneys causing structural damage [24, 25]. This interaction between the two organs is mainly carried out by systemic mediators such as serum cytokines [21, 26]. Our study sheds particular light on this aspect because it identifies an association between a trajectory with high first proteinuria level that increased during the first 3 days, and ICU patient mortality, independently of AKI. It is therefore likely that the death of these patients was due to organ failures other than renal failure, which could have activated renal inflammatory pathways thereby leading to a specified proteinuria trajectory. Identifying the mediators in the organ cross-talk during an acute phase of severe inflammation, is an essential step in the pathophysiological understanding of the multiple organ dysfunction syndrome, which is the cause of most deaths in the ICU.

The results of our study show an association between cancer and the trajectory 4, which is associated with an increased mortality. Among the deceased patients in our ICU, those with a cancer received no a particular management that could have led to a bias. In the literature, the high proteinuria levels associated with cancer are commonly due to glomerular lesions. Membranous nephropathy is the most common glomerulopathy associated with cancer [27]. Membranous glomerulonephritis is often associated with AKI and hypertension, and requires an immunosuppressive treatment. In our study, the trajectories were not associated with AKI and no patient was treated with immunosuppressive therapy for renal disease. The rapid change in the magnitude of proteinuria is suggestive of functional injury rather than histological glomerular lesions. The minimal change disease induces a nephrotic syndrome caused by glomerular function injury. In this syndrome, the proteinuria results from increased permeability of the glomerular filtration barrier induced by podocyte dysfunction [28]. This podocyte injury, which in turn, is due to circulating factors, mainly secreted by abnormal T cells. We hypothesized that an acute major inflammatory states such as sepsis or shock, could promote glomerular dysfunctions via circulating factors and lead to proteinuria changes. These circulating factors could induce or contribute to severe organ dysfunctions resulting in death. The cancer could promote the production of these circulating factors. The lymphocytes widely involved in the pathophysiology of minimal change disease [29] and in the modulation of immune response in sepsis [30] and cancer [31] could be a keystone in the mechanism of our findings.

Our study has several limitations. First, the proteinuria values associated with oligo-anuria, defined as the urine output less than 100ml/24h, were excluded from the statistical analyses because the urinary sediment analysis is unreliable when the urine output is low. Patients with oligo-anuria have severe AKI. Although we did not exclude these patients from analysis, only the proteinuria values related to the oligo-anuria period, we do not rule out a selection bias. Second, the study design did not explore the nature of proteinuria. To document this nature, a urine protein electrophoresis should be performed for each proteinuria. However, proteinuria greater than 1g/24h results mainly from glomerular damage and is only exceptionally of tubule-interstitial origin. The high proteinuria levels of trajectories 3 and 4 suggest the occurrence of glomerular injury. Proteinuria resulting from glomerular lesions, are plasma proteins with a high molecular weight and composed mainly of albumin. Only a prospective study could provide data on the nature of proteinuria. Third, the trajectory 2, which was characterized by low proteinuria, the clinical relevance of which is questionable, was also associated with increased mortality. This result indicates that not all severe inflammatory states resulting in death induce kidney damage with clinically relevant proteinuria. Trajectory 4 represented less than 10% of patients with severe inflammatory state. This minority group in the ICU patient population is therefore an argument for early identification of the distinct proteinuria trajectories in order to determine the target populations for mechanistic or therapeutic studies. Fourth, our results were expressed as proteinuria trajectories without providing a specified threshold for proteinuria associated with high mortality. It seems more reasonable to us to identify patterns of trajectories than thresholds in line with the same argument that a change in serum creatinine rather than a threshold should be used to define AKI [19]. This reasoning is supported by the results of our study, in which there was no difference between trajectories 3 and 4 in the first proteinuria measurement of the ICU stay. However, there was a great difference in change and in the association with mortality. In addition, assessment of a change in proteinuria rather than a threshold limits the bias caused by proteinuria secondary to chronic glomerular renal diseases, which have no reason to vary, except in cases of glomerulopathy flare-ups. Such cases were not found in our study population. Fifth, we assessed the magnitude of proteinuria in g/24h and not with the proteinuria/urine creatinine ratio. This ratio can replace proteinuria in g/24h to assess the magnitude of proteinuria particularly in non-hospitalized patients. The proteinuria and urine creatinine used for this ratio can be measured from a urine sample at any time of the day. This ratio avoids urine collection over 24h, which is complicated and difficult with risks of false measurements when the patient does not have a urinary catheter [32]. However, no such constraints exist in the ICU. The gold standard to assess the magnitude of proteinuria is in terms of g/24h when sampling can be performed reliably, which is the case in the ICU [32]. The urine collection over 24h is easy in ICU patients with sepsis or shock because they have a urinary catheter. Finally, the absence of systematic measurements of proteinuria in all patients admitted to the ICU and the monocentric design of our study could have influenced our findings. In accordance with guideline recommendations, the measurements of proteinuria in our department, are most often associated with those of urine electrolytes [19] when there are elevated serum creatinine levels or electrolytes disorders. Sensitivity analysis is used to confirm the robustness of our results.

More studies with much larger datasets are needed to validate and expand our observations. To investigate the association between proteinuria and outcome in the ICU, it might be interesting to measure daily the proteinuria levels over 24h in patients with sepsis or shock during the first 3 ICU days and subsequently to perform one or two measurements between day 4 and day 10. In contrast to a first high proteinuria value, defined as close to or higher than 2g/24h that decreases during the first 3 days, a high first proteinuria value that increases during the first 3 days seems to be associated with increased mortality.

In conclusion, this exploratory study of ICU patients with sepsis or shock identified four proteinuria trajectories with distinct patterns of proteinuria change over time and mortality rates. In our study, a proteinuria trajectory characterized by high first proteinuria level, close to 2 g/24 h and that had increased in the first 3 days of the ICU stay, was associated with increased mortality, regardless of acute kidney injury. These results provide novel insights into renal pathophysiology in severe inflammatory conditions and may be helpful to investigate subphenotypes of kidney injury in relation to proteinuria change among ICU patients in future studies.

## Supporting information

S1 Checklist STROBE statement—checklist of items that should be included in reports of observational studies. (DOCX)

S1 Fig Flowchart of patients admitted to ICU with sepsis or shock. (DOCX)

S2 Fig Proteinuria trajectories for patients with at least three proteinuria measurements during the first 10 days in ICU. blue curve: trajectory 1; red curve: trajectory 2; green curve: trajectory 3; purple curve: trajectory 4.
(DOCX)

S3 Fig Proteinuria trajectories with data imputed by multiple imputation by the two-fold fully conditional specification algorithm during the first 10 days in ICU. blue curve: trajectory 1; red curve: trajectory 2; green curve: trajectory 3; purple curve: trajectory 4.
(DOCX)

S1 Table Prognostic factors of mortality (univariable analysis). (DOCX)

S2 Table Multivariable analysis between the trajectories 3 and 4. (DOCX)

## List of abbreviations

AKI: Acute Kidney Injury
BMI: Body Mass Index
CI: Confidence Intervals
ICU: Intensive Care Unit
RRT: Renal replacement therapy
RR: Relative Risk
SAPS: Simplified Acute Physiology Score
