# Supplementary figures and images for: Association between proteinuria trajectories and outcomes in critically ill patients with sepsis or shock
Source: PLoS One. 2022 Aug 24;17(8):e0272835. doi: 10.1371/journal.pone.0272835 (PMC9401181; doi:10.1371/journal.pone.0272835)

**Supporting information**

**Figure S1: Flowchart of patients admitted to ICU with sepsis or shock**


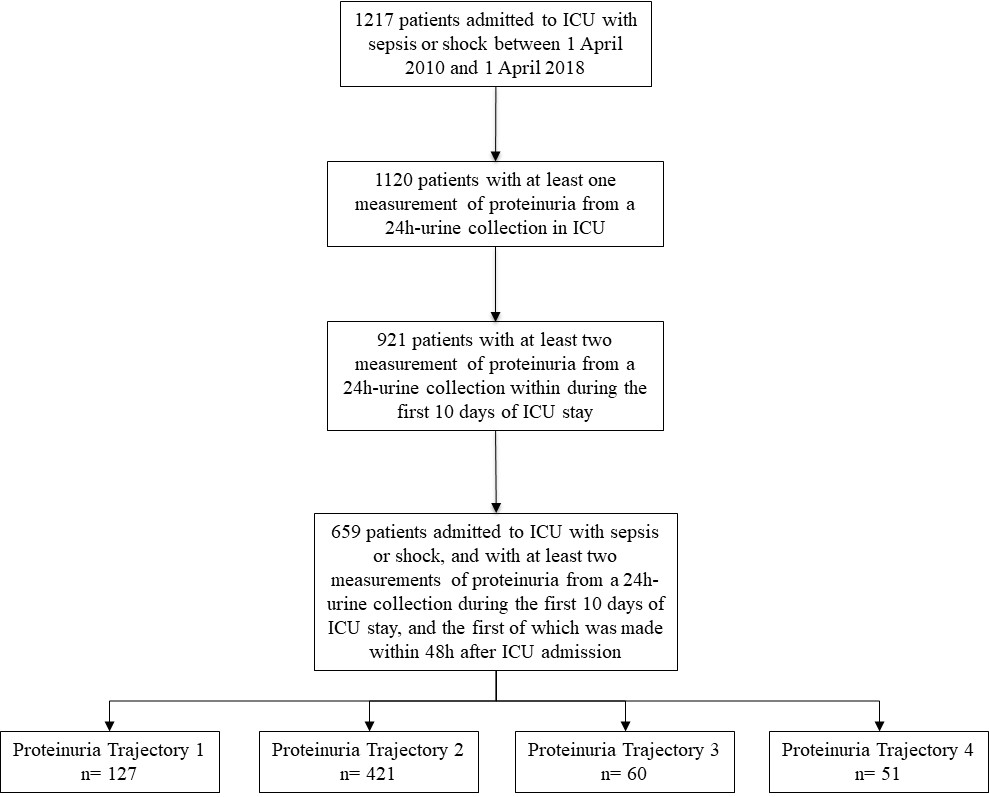

Supplement: S1 Fig — (DOCX) [file pone.0272835.s002.docx]
