# Supplementary material for: Association between proteinuria trajectories and outcomes in critically ill patients with sepsis or shock
Source: PLoS One. 2022 Aug 24;17(8):e0272835. doi: 10.1371/journal.pone.0272835 (PMC9401181; doi:10.1371/journal.pone.0272835)

**Supporting information**

**Figure S3: Proteinuria trajectories with data imputed by multiple imputation by the two-fold fully conditional specification algorithm during the first 10 days in ICU**


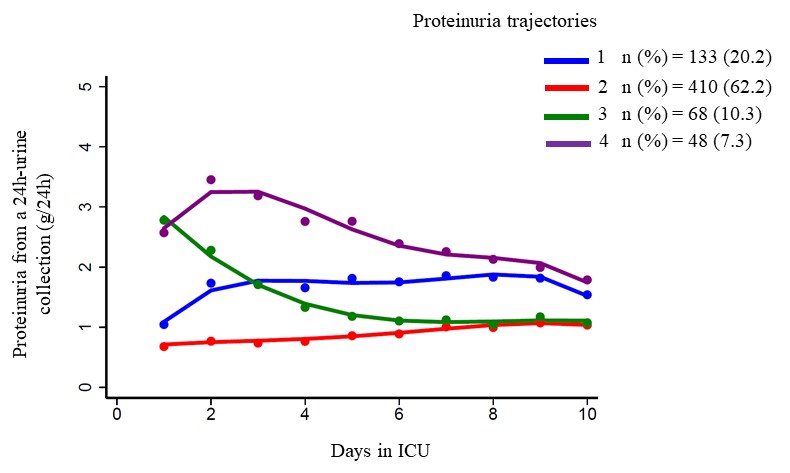

Supplement: S3 Fig — blue curve: trajectory 1; red curve: trajectory 2; green curve: trajectory 3; purple curve: trajectory 4. (DOCX) [file pone.0272835.s004.docx]
