# Supplementary material for: Association between proteinuria trajectories and outcomes in critically ill patients with sepsis or shock
Source: PLoS One. 2022 Aug 24;17(8):e0272835. doi: 10.1371/journal.pone.0272835 (PMC9401181; doi:10.1371/journal.pone.0272835)
Supplement: S1 Table — (DOCX) [file pone.0272835.s005.docx]

**Table S1**: **Prognostic factors of mortality (Univariable analysis)**

| Variables | HR 95%CI | p-value |
| --- | --- | --- |
| Male gender, n (%) | 1.48 [1.07-2.04] | 0.02 |
| Age, years | 1.02 [1.01-1.04] | <0.001 |
| Chronic kidney disease, n (%) | 0.84 [0.41-1.70] | 0.63 |
| Hypertension, n (%) | 0.73 [0.49-1.11] | 0.14 |
| Chronic heart failure, n (%) | 1.14 [0.75-1.72] | 0.54 |
| Cancer, n (%) | 2.01 [1.45-2.78] | <0.001 |
| Cirrhosis, n (%) | 1.43 [0.89-2.30] | 0.14 |
| Diabetes, n (%) | 1.11 [0.68-1.81] | 0.67 |
| Renin-angiotensin-aldosterone system blockers, n (%) | 1.01 [0.55-1.85] | 0.98 |
| Weight at ICU admission, kg | 1.0 [0.99-1.01] | 0.63 |
| BMI at ICU admission, kg/m² | 1.0 [0.98-1.02] | 0.56 |
| SAPS II at ICU admission, points | 1.04 [1.03-1.07] | <0.001 |
| Major inflammatory state, n (%) |  |  |
| Septic shock | 1.83 [1.08-3.07] | 0.02 |
| Cardiogenic shock | 2.37 [1.25-4.47] | 0.008 |
| Hypovolemic/Haemorrhagic shock | 1.31 [0.72-2.36] | 0.38 |
| Sepsis | ref |  |
| Invasive mechanical ventilation, n (%) | 2.04 [1.30-3.21] | 0.002 |
| Non-invasive mechanical ventilation, n (%) | 0.80 [0.60-1.06] | 0.12 |
| Vasopressor drugs, n (%) | 3.69 [2.01-6.78] | <0.001 |
| Inotropic drugs, n (%) | 2.32 [1.71-3.14] | <0.001 |
| Diuretics in ICU, n (%) | 1.01 [0.74-1.35] | 0.99 |
| Serum creatinine at day 1, µmol/L | 1.27 [1.04-1.57] | 0.02 |
| Serum creatinine at day 10, µmol/L | 0.96 [0.51-1.83] | 0.91 |
| Acute kidney injury, n (%) |  |  |
| Stage 1 | 0.79 [0.53-1.19] | 0.26 |
| Stage 2 | 0.66 [0.40-1.22] | 0.19 |
| Stage 3 | 1.50 [1.06-2.12] | 0.02 |
| Acute kidney injury requiring renal replacement therapy, n (%) | 2.17 [1.64-2.88] | <0.001 |
| Minimum level of PO_2_/FiO_2_ ratio during the first 10 days | 0.84 [0.65-1.11] | 0.23 |
| Maximum level of total bilirubin during the first 10 days, µmol/L | 1.24 [1.08-1.43] | 0.002 |
| First proteinuria, g/24h | 0.92 [0.79-1.08] | 0.31 |
