# Supplementary material for: Association between proteinuria trajectories and outcomes in critically ill patients with sepsis or shock
Source: PLoS One. 2022 Aug 24;17(8):e0272835. doi: 10.1371/journal.pone.0272835 (PMC9401181; doi:10.1371/journal.pone.0272835)
Supplement: S2 Table — (DOCX) [file pone.0272835.s006.docx]

**Table S2:** **Multivariate analysis between the trajectories 3 and 4***

| **Variable** | RR [95% CI] | p-value |
| --- | --- | --- |
| Age | 1.00 [0.97–1.03] | 0.98 |
| Male gender | 1.23 [0.40–3.77] | 0.71 |
| Cancer | 8.91 [2.09–38.02] | <0.001 |
| Cirrhosis | 0.32 [0.03–3.24] | 0.33 |
| Inotropic drugs | 0.17 [0.04–0.69] | 0.01 |
| Acute kidney injury, stage 1 | 1.19 [0.31–4.49] | 0.80 |
| Acute kidney injury, stage 2 | 0.79 [0.17–3.73] | 0.77 |
| Acute kidney injury, stage 3 | 1.83 [0.46–7.20] | 0.39 |

**adjusted on the first proteinuria.*
